# Complete mitochondrial genome and phylogenetic analysis of the Gray’s pipefish *Halicampus grayi* Kaup 1856

**DOI:** 10.1080/23802359.2019.1681915

**Published:** 2019-10-24

**Authors:** Jianzhen Chen, Mengru Lai, Siya Sun, Guangji Zhang, Yuqing Ge, Rubin Cheng

**Affiliations:** a College of Pharmaceutical Science, Zhejiang Chinese Medical University, Hangzhou, Zhejiang, P. R. China;; b The First Affiliated Hospital, Zhejiang Chinese Medical University, Hangzhou, Zhejiang, P. R. China

**Keywords:** *Halicampus grayi*, complete mitochondrial genome, phylogenetic analysis

## Abstract

In the present study, the complete mitochondrial genome of *H. grayi* was determined and annotated. The circular mitogenome is 16,959 bp in length and contains 13 protein-coding genes (PCGs), two rRNA genes, 22 tRNA genes, and a control region. Most of the PCGs start with ATG, but *CO1* begins with a GTG start codon. Phylogenetic analysis revealed that* H. grayi* was strictly related to network pipefish *Corythoichthys flavofasciatus* with 100% bootstrap support value. This work provides basic molecular information that would be useful for further investigation on conservation genetics and evolutionary relationships of *H. grayi*.

The Gray’s pipefish *Halicampus grayi* (Kaup, 1856) is a widespread Indo-Pacific species that inhabits muddy sand, estuaries, and coral reefs with a maximum depth of 100 m (Dawson 1982). The species is one of the most important resources for syngnathids which have been traded heavily throughout the region for traditional medicines and the aquarium trade (Vincent et al. 2011). Due to the strong market demand of dried pipefish, the wild-caught specimens of *H. grayi* were often traded as main substitutions for Chinese medicinal pipefishes (Gao et al. 2018). Furthermore, partial mitochondrial DNA barcoding markers of *16S* and *COI* genes failed to distinguish certain pipefishes as distinct species (Garcia et al. 2019). The Complete mitochondrial genome of Gray’s pipefish could provide the basic molecular data and contribute to the development of molecular identification and further conservation strategy (Chen et al. 2018; Zhu et al. 2019).

In this study, we sequenced the complete mitochondrial genome of *H. grayi* and investigated the phylogenetic relationship of Genus Halicampus among Syngnathidae species. The specimen of *H. grayi* was collected from Bozhou herbal medicine market of Anhui Province and identified according to the typical morphological characteristics including the relative short snout and large eyes. The sample of *H. grayi* GSH-06 was deposited in the collection centre of Zhejiang Chinese Medical University. The entire mtDNA of this pipefish species was sequenced by universal primers according to the previous researches (Ge et al. 2018; Lai et al. 2019). The complete mtDNA sequence of *H. grayi*, with the annotated genes, was submitted to GenBank with the accession number of MN064721.

The complete mitochondrial genome of *H. grayi* was 16,959 bp in length with the overall base compositions of 30.03, 29.25, 24.54, and 16.17% for A, T, C, and G, respectively. The high A + T content bias (59.28%) was basically consistent with those of other syngnathid fishes (Wang et al. 2019). The mitogenome of *H. grayi*, in total, contained 13 protein-coding genes (PCGs), 22 tRNA genes, 2 rRNA genes, and a control region. Among these genes, all PCGs genes have a typical ATG initiation codon except *CO1* which starts with GTG. The complete stop codon TAA was found in seven PCGs (*CO2*, *ATP8*, *ATP6*, *CO3*, *ND4L*, *ND5*, and *ND6*), and the remaining six genes terminated with incomplete TA or T. The 22 tRNA genes ranged from 67 to 74 bp in length with the typical clover-leaf structure except *tRNA-Ser* (AGC) which lacks the dihydrouridine arm. The control region was 1126 bp in length which was significantly longer than that in other Syngnathidae mitogenomes (Fang et al. 2018; Sun et al. 2019).

The phylogenetic tree of *H. grayi* and other Syngnathidae species were constructed by maximum-likelihood (ML) method based on the complete mitochondrial protein coding sequences. As showing in Figure 1, *H. grayi* and *Corythoichthys flavofasciatus* clustered together with high statistical support, indicating a close genetic relationship. Furthermore, the Gray’s pipefish *H. grayi* shared obviously high nucleotide sequence identity (99.45%) with the network pipefish *C. flavofasciatus*, suggesting further taxonomic revision among the two syngnathid species (Zhang et al. 2015). The genera Halicampus, Corythoichthys, and Trachyrhamphus clustered into a monophyletic group with high bootstrap values, which is sister to the clade clustered by Hippocampus species. These results suggested that fishes in the three genera could be developed as potential alternatives to seahorse species. This study provides basic molecular data of *H. grayi*, which was important for the development of specific DNA barcodes and understanding of evolutionary history and conservation strategy in Family Syngnathidae.

**Figure 1.**
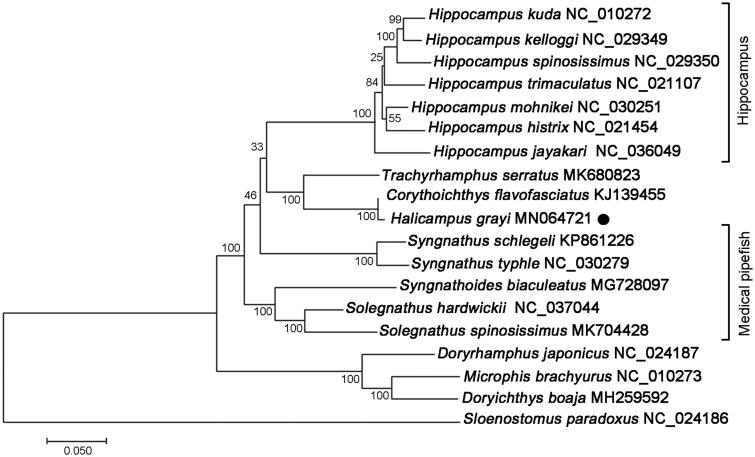
ML phylogenetic tree of *Halicampus grayi* and other representative Syngnathidae species based on the complete mitochondrial protein coding sequences. Numbers above the lines indicate the bootstrap value for the ML analysis based on 100 replicates. The GenBank accession numbers were listed following the species name.
